# Supplementary material for: Dynamic Electron Correlation Energy for Multireference Wave Function Methods from One- and Two-Electron Reduced Density Matrices
Source: J Chem Theory Comput. 2026 Sep 11;22(18):9215–34. doi: 10.1021/acs.jctc.6c01013 (PMC13602902; doi:10.1021/acs.jctc.6c01013)
Supplement: Supplementary file 1 [file ct6c01013_si_001.pdf]

Supplementary Material for Dynamic electron correlation energy for multireference wavefunction methods from one- and two-electron reduced density matrices

Michał Hapka,[†] Aleksandra Tucholska,[‡] and Katarzyna Pernal[*,¶]

†University of Warsaw, Faculty of Chemistry, ul. L. Pasteura 1, 02-093 Warsaw, Poland

‡J. Heyrovský Institute of Physical Chemistry, Academy of Sciences of the Czech Republic, 18223 Prague 8 Czech Republic

¶Institute of Physics, Lodz University of Technology, ul. Wolczanska 217/221, 93-005 Lodz, Poland

E-mail: pernalk@gmail.com

Abstract

Contents

S1 Biradicals . . . 2

S1.1 Computational details . . . 2

S1.2 Absolute Energies of Singlet and Triplet states and Singlet–Triplet Gaps 2

S1.3 Active spaces . . . 4

S1.4 Optimized Molecular Geometries for Biradicals . . . 4

S2 Excited states **7**
S2.1 Computational details 7
S2.2 Errors 8
S2.3 Error box plots 9

S3 Iron Complexes **10**
S3.1 Computational details 10
S3.2 Orbital visualization 10
S3.3 Absolute energies of spin states HS-LS Energy Differences of iron complexes 13
S3.4 Optimized Geometries of Iron Complexes 15

References **16**

S1 Biradicals

S1.1 Computational details

The CASSCF calculations for the biradical set (molecules from Ref. 1) were performed with the PySCF[2–4] package. State-specific CASSCF wavefunctions were computed for the singlet and triplet states separately, using the active spaces described below and the maug-cc-pVTZ basis set.[5] The SC-NEVPT2[6] correlation energies were computed using the PySCF implementation. The resulting CASSCF one- and two-particle density matrices were subsequently used for post-CASSCF calculations in the GAMMCOR[7] code, including the AC0 (in the ffAC0, phAC0 and ppAC0 variants), srPBE, spin-srPBE, srPDFT, and PDFT calculations. DEA-EOMCC(4p–2h) data from Ref. 8 is taken as an accurate reference.

S1.2 Absolute Energies of Singlet and Triplet states and Singlet–Triplet Gaps

Table S1: Absolute energies (in Ha) for ground singlet (S) state and triplet (T) state. Singlet-triplet (ΔST) energy differences (in eV) obtained with methods used in work. Signed error with respec to accurate DEA-EOMCC(4p–2h) data (acc.) taken from Ref. 8

System	acc.	Mult.	CASSCF	AC0	srDFT	spin-srDFT	srPDFT	PDFT	NEVPT2
C4H4	0.18	S	-153.748410	-154.371338	-154.385811	-154.385811	-154.426131	-154.469377	-154.378766
		T	-153.732130	-154.366007	-154.381068	-154.365178	-154.417718	-154.469899	-154.377607
		ΔST	0.44	0.15	0.13	0.56	0.23	-0.01	0.03
		error	0.26	-0.04	-0.05	0.38	0.05	-0.20	-0.15
C5H5+	-0.60	S	-192.005403	-192.760048	-192.797468	-192.797468	-192.828897	-192.889883	-192.772601
		T	-192.029390	-192.785249	-192.818700	-192.837405	-192.853036	-192.915960	-192.803018
		ΔST	-0.65	-0.69	-0.58	-1.09	-0.66	-0.71	-0.83
		error	-0.05	-0.08	0.02	-0.48	-0.05	-0.11	-0.23
C4H3NH2	0.11	S	-208.802069	-209.654574	-209.693276	-209.693276	-209.731565	-209.783555	-209.661920
		T	-208.787737	-209.652278	-209.690996	-209.711597	-209.725376	-209.786757	-209.663750
		ΔST	0.39	0.06	0.06	-0.50	0.17	-0.09	-0.05
		error	0.28	-0.05	-0.05	-0.61	0.05	-0.20	-0.16
C4H3CHO	0.16	S	-266.519508	-267.546224	-267.598706	-267.644935	-267.636852	-267.702259	-267.553550
		T	-266.504407	-267.542415	-267.594922	-267.614854	-267.629455	-267.703707	-267.553212
		ΔST	0.41	0.10	0.10	0.82	0.20	-0.04	0.01
		error	0.25	-0.05	-0.06	0.66	0.04	-0.20	-0.15
C4H2NH2CHO	0.25	S	-321.582453	-322.845091	-322.922625	-322.922625	-322.949046	-323.039587	-322.848892
		T	-321.560421	-322.834885	-322.911262	-322.930494	-322.941957	-323.027280	-322.845634
		ΔST	0.60	0.28	0.31	-0.21	0.19	0.33	0.09
		error	0.35	0.03	0.06	-0.46	-0.05	0.09	-0.16
C4H2-1,2-$(CH_2)_2$	3.37	S	-230.755039	-231.709178	-231.741383	-231.741383	-231.782504	-231.853490	-231.717489
		T	-230.632652	-231.579023	-231.605277	-231.626506	-231.659613	-231.726977	-231.593769
		ΔST	3.33	3.54	3.70	3.13	3.34	3.44	3.37
		error	-0.04	0.17	0.34	-0.24	-0.02	0.07	0.00
C4H2-1,3-$(CH_2)_2$	-0.80	S	-230.660253	-231.613374	-231.642429	-231.642429	-231.689901	-231.765261	-231.624473
		T	-230.694204	-231.644689	-231.667917	-231.699111	-231.718621	-231.789332	-231.655724
		ΔST	-0.92	-0.85	-0.69	-1.54	-0.78	-0.65	-0.85
		error	-0.12	-0.05	0.11	-0.74	0.02	0.15	-0.05
MUE			0.19	0.07	0.10	0.51	0.04	0.15	0.13
MSE			0.13	-0.01	0.05	-0.21	0.01	-0.06	-0.13
MAX ERROR			0.35	0.17	0.34	0.74	0.05	0.20	0.23
MU%E			103.25	19.70	23.09	220.49	20.67	69.08	61.59

S1.3 Active spaces

The CASSCF active spaces included all valence π orbitals of the carbon atoms belonging to or adjacent to the ring.[9] For comparison, the πCPO active-space scheme introduced in Ref. 1 employs the following orbital spaces: CAS(4,6) for C_4H_4, $C_5H_5^+$, $C_4H_3NH_2$, C_4H_3CHO, and $C_4H_2NH_2CHO$, and CAS(6,8) for the isomers C_4H_2-1,2-$(CH_2)_2$ and C_4H_2-1,3-$(CH_2)_2$.

Table S2: Complete active space (CAS) definitions and natural orbital occupation numbers obtained from CASSCF calculations.

Molecule	Active space	State	Natural orbital occupations
C4H4	CAS(4,4)	S	1.90, 1.00, 1.00, 0.10
		T	1.91, 1.00, 1.00, 0.09
C5H5+	CAS(4,5)	S	1.91, 1.56, 0.44, 0.02, 0.07
		T	1.92, 1.00, 1.00, 0.04, 0.04
C4H3NH2	CAS(4,4)	S	0.95, 0.50, 0.50, 0.05
		T	0.96, 0.50, 0.50, 0.04
C4H3CHO	CAS(4,4)	S	1.90, 1.07, 0.93, 0.10
		T	1.91, 1.00, 1.00, 0.09
C4H2NH2CHO	CAS(4,4)	S	0.98, 0.94, 0.06, 0.02
		T	0.96, 0.50, 0.50, 0.04
C4H2-1,2-$(CH_2)_2$	CAS(6,6)	S	1.95, 1.91, 1.89, 0.09, 0.11, 0.05
		T	1.92, 1.77, 1.15, 0.24, 0.85, 0.07
C4H2-1,3-$(CH_2)_2$	CAS(6,6)	S	1.94, 1.93, 1.43, 0.57, 0.05, 0.09
		T	1.94, 1.88, 1.00, 1.00, 0.06, 0.12

S1.4 Optimized Molecular Geometries for Biradicals

Geometries taken from Ref 10. The Cartesian coordinates in Å of seven biradical systems employed in the calculations.

```
C4H4
8
C        0.000000    1.026841    0.000000
C        1.026841    0.000000    0.000000
C        0.000000   -1.026841    0.000000
C       -1.026841    0.000000    0.000000
```

H	0.000000	2.118166	0.000000
H	2.118166	0.000000	0.000000
H	0.000000	-2.118166	0.000000
H	-2.118166	0.000000	0.000000

C5H5+

C	0.000000	1.218676	0.000000
C	1.159030	0.376592	0.000000
C	0.716320	-0.985930	0.000000
C	-0.716320	-0.985930	0.000000
C	-1.159030	0.376592	0.000000
H	0.000000	2.311326	0.000000
H	2.198201	0.714239	0.000000
H	1.358563	-1.869902	0.000000
H	-1.358563	-1.869902	0.000000
H	-2.198201	0.714239	0.000000

C4H3NH2
10

C	-0.038842	0.667358	0.013902
C	-0.069293	-0.744519	1.496778
C	-0.093175	0.704750	1.455243
C	-0.019711	-0.775095	0.047746
H	-0.055984	-1.492092	2.283548
H	-0.140810	1.501553	2.196308
H	0.225925	2.536881	-0.720770
H	0.284590	1.322053	-1.875949
H	0.010883	-1.555606	-0.711331
N	-0.111291	1.614637	-0.985894

C4H3CHO
10

C	0.120170	-1.528913	-1.196781
C	0.034538	-0.617525	-0.067594
C	-0.066917	-0.734343	1.394492
C	-0.084239	0.697111	1.491929
C	0.014554	0.843001	0.065222
H	0.110078	-2.614798	-0.937472
H	-0.110658	-1.559580	2.106439
H	-0.147771	1.400027	2.325959

```
H          0.057411        1.677460       -0.634427
O          0.198782       -1.148509       -2.359053

C4H2NH2CHO
12
C          0.508276        0.548783        0.140341
C         -0.453143       -0.514797       -0.120616
C         -0.659160       -0.684423        1.328768
C          0.311523        0.388020        1.546942
C         -0.894389       -1.049891       -1.387035
H         -1.267485       -1.323931        1.963483
H          0.727466        0.878128        2.430626
H         -1.646940       -1.871474       -1.359181
H          1.878883        2.023354       -0.352163
H          1.142331        1.164654       -1.691261
N          1.241858        1.321176       -0.696744
O         -0.462507       -0.617775       -2.459648

C4H2-1,2-(CH2)2
12
C      1.708581   -1.087143    0.000000
C     -1.708581   -1.087143    0.000000
C      0.755922   -0.161742    0.000000
C     -0.755922   -0.161742    0.000000
C     -0.677500    1.320688    0.000000
C      0.677500    1.320688    0.000000
H      2.764177   -0.812830    0.000000
H      1.458640   -2.148661    0.000000
H     -1.458640   -2.148661    0.000000
H     -2.764177   -0.812830    0.000000
H     -1.431821    2.106540    0.000000
H      1.431821    2.106540    0.000000

C4H2-1,3-(CH2)2
12
C     -2.397122    0.000000    0.000000
C      2.397122    0.000000    0.000000
C      0.000000   -1.026676    0.000000
C      1.036908    0.000000    0.000000
C      0.000000    1.026676    0.000000
C     -1.036908    0.000000    0.000000
```

```
H    -2.957776   -0.934934    0.000000
H    -2.957776    0.934934    0.000000
H     2.957776    0.934934    0.000000
H     2.957776   -0.934934    0.000000
H     0.000000   -2.114833    0.000000
H     0.000000    2.114833    0.000000
```

S2 Excited states

S2.1 Computational details

The excited-state benchmark covers the singlet and triplet vertical excitation energies from Thiel's set,[11] comprising 28 closed-shell organic chromophores. Geometries, active spaces and basis sets follow Ref. 11. The def2-TZVP basis[12] was used throughout. All molecular geometries are attached in a separate file (`geoms.dat`).

CASSCF references. State-specific (SS) and state-averaged (SA) CASSCF calculations were performed with the PySCF[2–4] package. The complete list of active-space sizes, the distribution of active orbitals across irreducible representations, and the set of states included in the state-averaging for each molecule are collected in the spreadsheet `supporting_information_singlets.xlsx` (for singlets) and `supporting_information_triplets.xlsx` (for triplets), in the tab `active_space`.

Post-CASSCF dynamic correlation: AC0, srPBE, srPDFT, PDFT. The CASSCF one- and two-particle reduced density matrices were exported from PySCF and used in subsequent post-CASSCF calculations performed with the Gammcor code,[7] including srDFT, srPDFT, PDFT and the AC0 variants (phAC0, ppAC0, ffAC0). The range-separation parameter $\mu = 0.4$ bohr^{-1} was employed in all range-separated calculations. Excitation energies were obtained both in a state-specific manner (energy difference between two SS-CASSCF references) and in a state-averaged manner (energy difference between two roots of the same SA-CASSCF calculation). For triplet excitation energies, a spin-density-dependent variant of the short-range exchange-correlation energy, $E_{\mathrm{xc}}^{\mathrm{SR}}[\rho_C, \rho_S]$ (denoted spin-srDFT), was evaluated in addition. All results are collected in `supporting_information_singlets.xlsx` and `supporting_information_triplets.xlsx`; each spreadsheet contains absolute total energies in Hartree (worksheet `Abs. Energy (Ha)`), vertical excitation energies in eV (`Exc. Energy (eV)`), signed errors with respect to the CC3 reference values[11,13,14] (`Errors`), per-method statistical metrics (`Summary`), active-space specifications (`Active Space`), and a description of each method including key computational details (`Methods`).

Self-consistent SCF-srDFT calculations. Self-consistent MC-srDFT calculations were carried out with the DALTON[15] program (release Dalton2022.0) using the same def2-TZVP basis set and active spaces as in the post-CASSCF calculations, with $\mu = 0.4$ bohr^{-1}. Two short-range exchange–correlation functionals were employed: the srPBE functional[16,17] (SRXPBEGWS/SRCPBEGWS in Dalton; referred to as SCF-srPBE) and the srLDA functional[18] (SCF-srLDA). Excitation energies were obtained as state-specific differences between two independent SCF-srDFT calculations targeting the ground and the excited state. The results are collected in the singlet/triplet are tabulated in `supporting_information_singlets.xlsx` and `supporting_information_triplets.xlsx`.

S2.2 Errors

Table S3: Statistical analysis of 140 singlet vertical excitation energies obtained with methods considered in this work in the def2-TZVP[12] basis set. Errors are reported with respect to CC3 reference values.[11,13,14] NEVPT2-SS-PC results calculated in Dalton.[15] NEVPT2-SA-PC/1 and CASPT2 results from Schapiro et al.[13] All values in eV.

System	Stat.	CASSCF	srDFT	srPDFT	PDFT	AC0	NEVPT2
SS_singlet							
	MUE	0.80	0.38	0.19	0.61	0.26	0.32
	MSE	0.72	0.00	-0.03	-0.55	0.16	-0.11
	SD	0.73	0.47	0.25	0.63	0.28	0.32
	MAX	2.40	1.49	0.83	2.11	0.99	1.91
SA_singlet							
	MUE	0.99	0.47	0.22	0.67	0.31	0.24
	MSE	0.94	-0.03	0.01	-0.57	0.20	-0.09
	SD	0.80	0.59	0.32	0.78	0.34	0.2
	MAX	2.82	2.17	1.62	3.71	1.86	0.92

Table S4: Statistical analysis of 71 triplet excitation energies from methods considered in this work in the def2-TZVP[12] basis set. Errors are relative to CC3.[11,14,19] NEVPT2-SS-PC from Dalton;[15] NEVPT2-SA-PC/1 and CASPT2 from Schapiro et al.[19] All values in eV.

System	Stat.	CASSCF	srDFT	spin-srDFT	srPDFT	PDFT	AC0	NEVPT2
SS_triplet								
	MAE	0.30	0.49	0.36	0.11	0.26	0.23	0.18
	MSE	0.11	0.37	-0.30	-0.01	-0.07	0.14	0.02
	SD	0.43	0.42	0.39	0.18	0.43	0.24	0.27
	MAX	1.19	1.21	1.79	0.51	1.78	0.09	1.09
SA_triplet								
	MAE	0.38	0.53	-	0.14	0.28	0.26	0.16
	MSE	0.26	0.38	-	0.03	-0.06	0.17	0.02
	SD	0.47	0.44	-	0.23	0.49	0.27	0.24
	MAX	1.34	1.21	-	0.99	1.87	1.10	0.96

S2.3 Error box plots

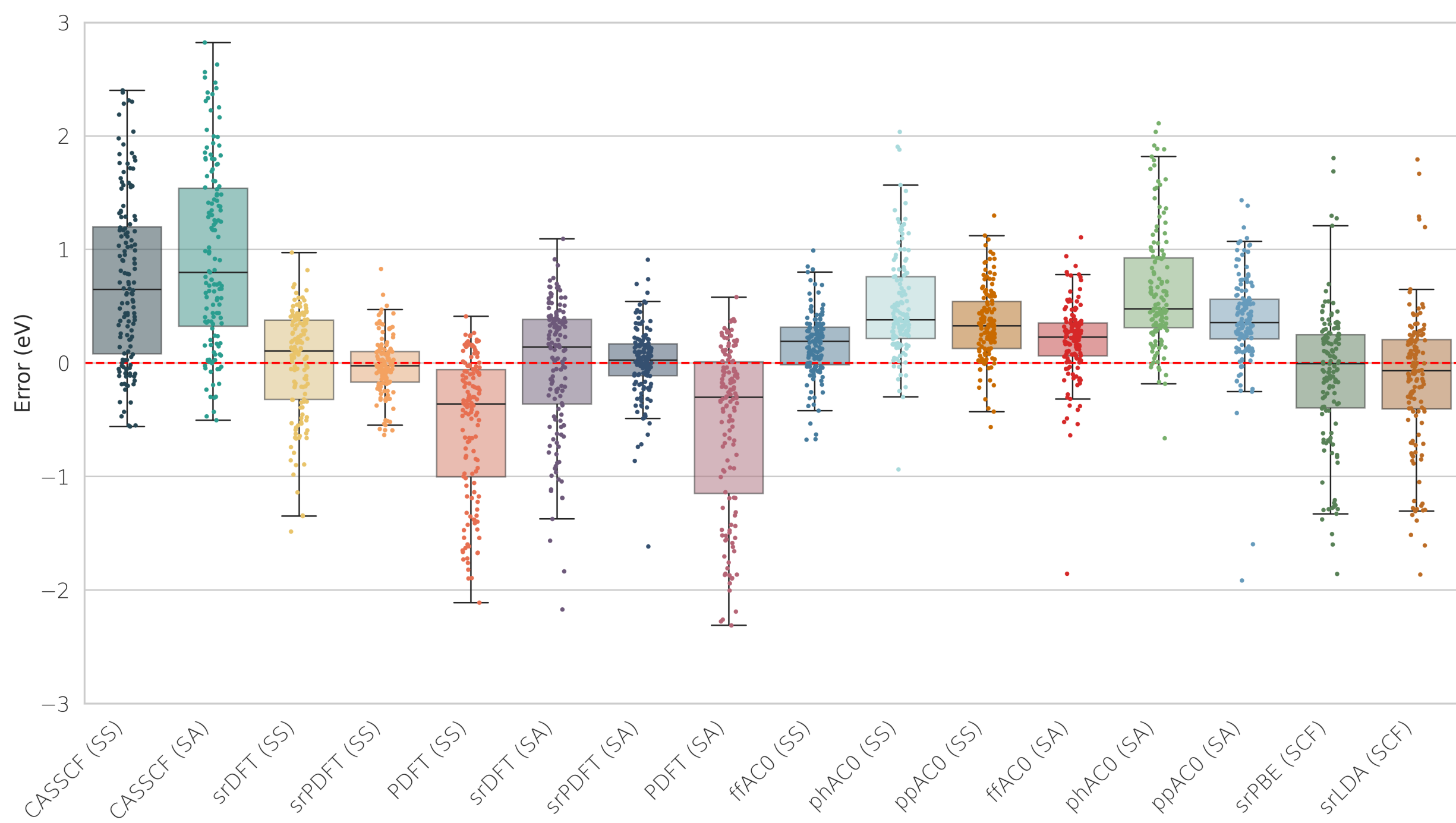

Figure S1: Error distribution for singlet excitation energies.

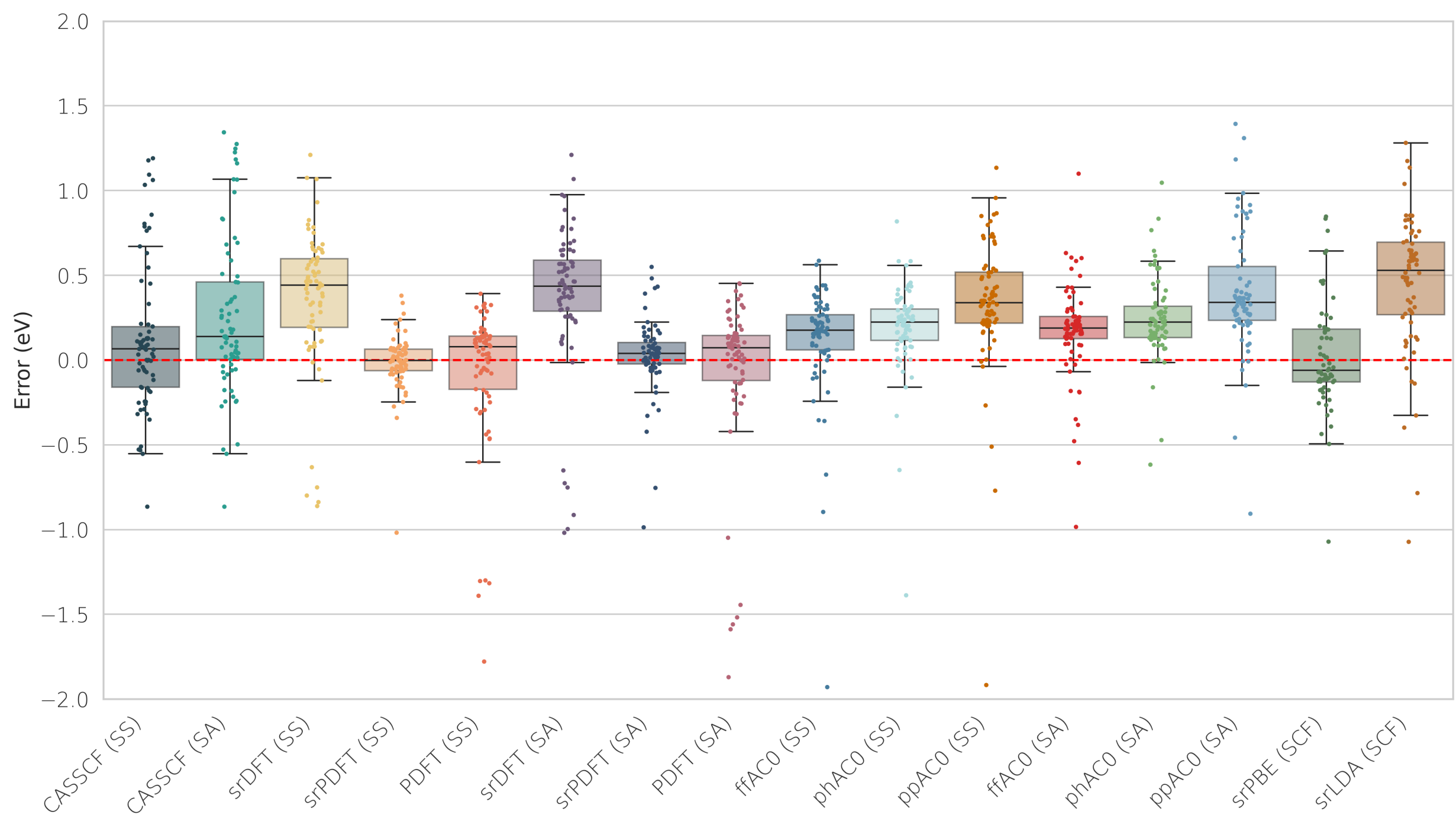

Figure S2: Error distribution for triplet excitation energies.

S3 Iron Complexes

S3.1 Computational details

The CASSCF calculations on iron complexes were performed using Molpro 2012.[20] The reference wavefunctions employed CAS(9,12) active space for $[\mathrm{Fe(H_2O)_6}]^{3+}$ and CAS(10,12) active space for $[\mathrm{Fe(NH_3)_6}]^{2+}$ and $[\mathrm{Fe(H_2O)_6}]^{2+}$. The cc-pwCVTZ-DK[21] for non-hydrogen atoms and cc-pVTZ-DK[22] for hydrogen atoms basis set was used throughout, with scalar relativistic effects treated via the Douglas–Kroll–Hess Hamiltonian (DKH2)[23] . The range-separation parameter $\mu = 0.4$ bohr^{-1} was used in all srDFT-based calculations. The resulting CASSCF one- and two-particle density matrices were subsequently used for post-CASSCF calculations in Gammcor[7] code, including the AC0 (ffAC0, ppAC0, phAC0 variants), srPBE, spin-srPBE, srPDFT, PDFT calculations.

S3.2 Orbital visualization

We present the molecular orbitals for the $\mathrm{Fe(H_2O)_6}^{3+}$ complex for high-spin and low-spin states. The active space comprise five Fe 3d orbitals (orange-pink), five Fe 4d orbitals (yellow-green), and two ligand orbitals responsible for σ bonding with the

Fe 3d shell (violet-pink). Orbital isosurfaces were rendered using molden files with IboView.[24,25]

Figure S3: Natural orbitals and their natural occupation numbers for $Fe(H_2O)_6^{3+}$ obtained from CASSCF calculations for the quartet state.

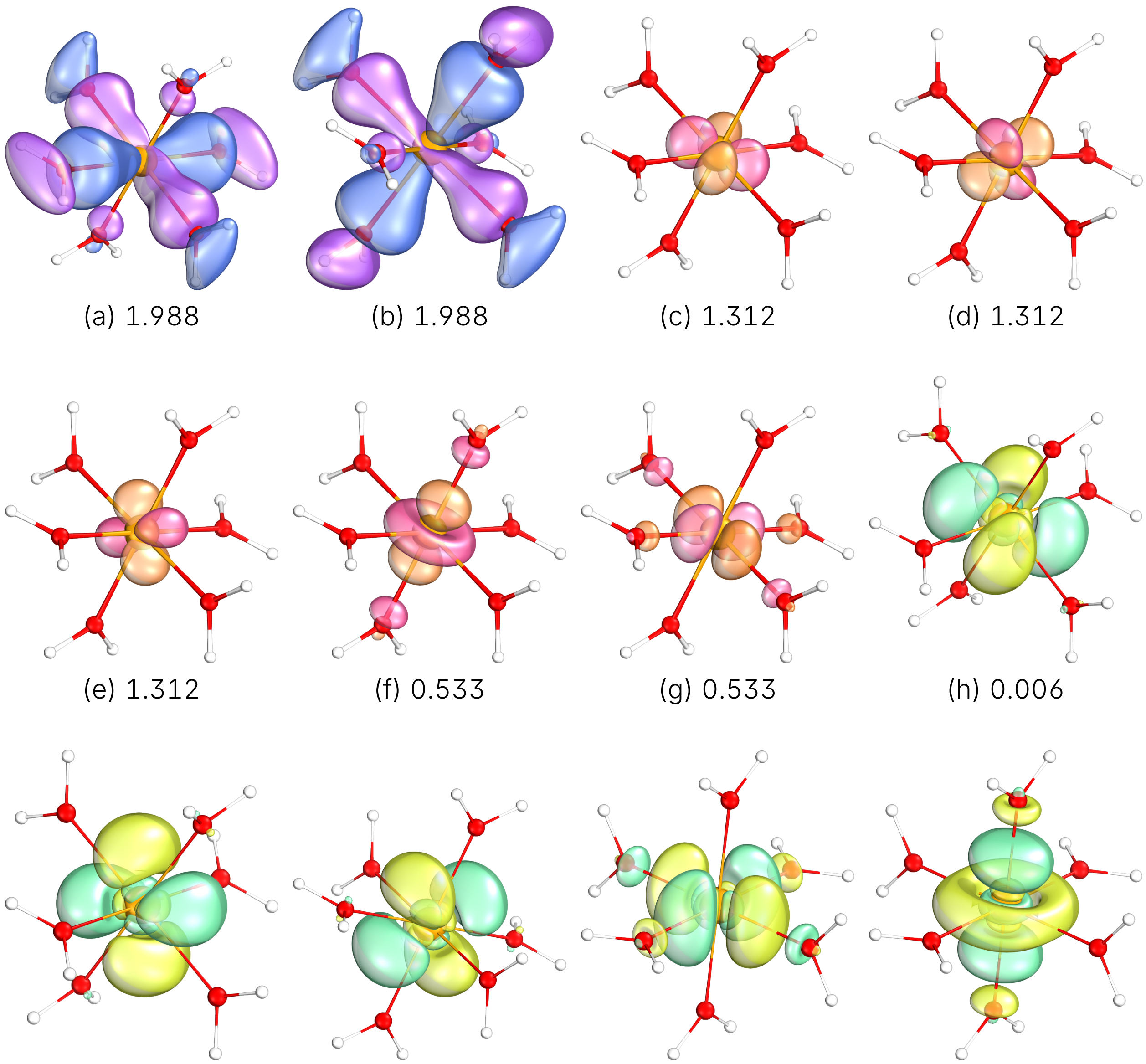

(a) 1.988 (b) 1.988 (c) 1.312 (d) 1.312

(e) 1.312 (f) 0.533 (g) 0.533 (h) 0.006

(i) 0.006 (j) 0.006 (k) 0.002 (l) 0.002

Figure S4: Natural orbitals and their natural occupation numbers for $Fe(H_2O)_6^{2+}$ obtained from CASSCF calculations for the sextet state.

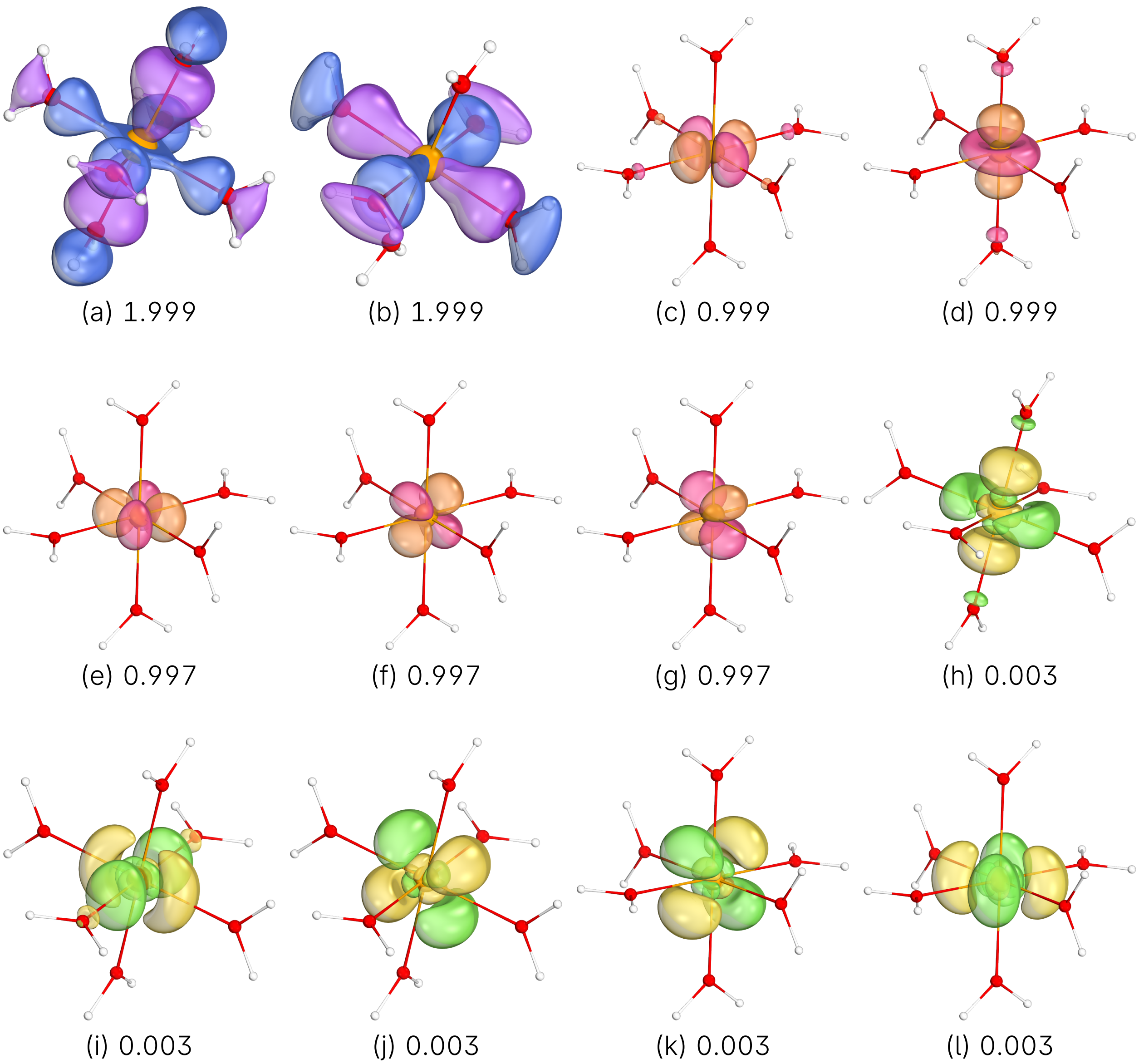

(a) 1.999 (b) 1.999 (c) 0.999 (d) 0.999

(e) 0.997 (f) 0.997 (g) 0.997 (h) 0.003

(i) 0.003 (j) 0.003 (k) 0.003 (l) 0.003

Table S5: Natural orbital occupation numbers from CASSCF calculations for Fe(III) and Fe(II) complexes.

Complex	Active space	State	Natural orbital occupations
$Fe(H_2O)_6^{3+}$	CAS(10,12)	Quartet	1.9882, 1.9882, 1.3122, 1.3122, 1.3122, 0.5328, 0.5328, 0.0057, 0.0057, 0.0057, 0.0022, 0.0022
		Sextet	1.9986, 1.9986, 0.9986, 0.9986, 0.9973, 0.9973, 0.9973, 0.0028, 0.0028, 0.0027, 0.0027, 0.0027
$Fe(H_2O)_6^{2+}$	CAS(10,12)	Quintet	1.9990, 1.9990, 1.3266, 1.3265, 1.3264, 0.9967, 0.9964, 0.0070, 0.0069, 0.0069, 0.0039, 0.0038
		Singlet	1.9978, 1.9973, 1.9528, 1.6446, 1.6443, 0.5168, 0.2111, 0.0118, 0.0099, 0.0098, 0.0026, 0.0012
$Fe(NH_3)_6^{2+}$	CAS(10,12)	Quintet	1.9990, 1.9990, 1.3256, 1.3256, 1.3256, 0.9968, 0.9967, 0.0078, 0.0077, 0.0077, 0.0042, 0.0042
		Singlet	1.9946, 1.9933, 1.9660, 1.6425, 1.6410, 0.5260, 0.1910, 0.0154, 0.0127, 0.0127, 0.0032, 0.0014

S3.3 Absolute energies of spin states HS - LS Energy Differences of iron complexes

Table S6: Absolute energies (in Ha) of spin states for Fe(III) and Fe(II) aqua and ammonia complexes. Si - singlet, Qt - quartet, Qu - quintet, Sx - sextet. High-spin (HS) –low-spin(LS) energy differences Δ_{HS-LS} (in kcal/mol) srPBE denotes the use of a charge density in the short-range functional, whereas spin-srPBE refers to the use of a spin-density functional.

Complex	Active space	Ref. kcal/mol	State	CASSCF	srPBE	spin-srPBE	srPDFT	PDFT	AC0	NEVPT2
$[Fe(H_2O)_6]^{3+}$	CAS (9,12)	-47.4 [26]	Sx	-1727.153075	-1729.658271	-1729.891947	-1729.885339	-1729.825967	-1729.767758	-1729.774946
			Qt	-1727.044237	-1729.725485	-1729.827067	-1729.835600	-1729.787534	-1729.692626	-1729.700537
			Δ_{HS-LS}	-68.30	42.20	-40.70	-31.20	-24.10	-47.10	-46.70
			Errors in kcal/mol	-20.9	89.6	6.7	16.2	23.3	0.3	0.7
			Errors in eV	-0.91	3.89	0.29	0.70	1.01	0.01	0.03
$[Fe(H_2O)_6]^{2+}$	SA-CAS (10,12)	-33.4 [27]	Qu	-1727.733365	-1730.346726	-1730.504344	-1730.508083	-1730.445359	-1730.370878	-1730.378534
			Si	-1727.640456	-1730.440599	-1730.440599	-1730.474660	-1730.414025	-1730.321177	-1730.325421
			Δ_{HS-LS}	-58.30	58.90	-40.00	-21.00	-19.70	-31.20	-33.30
			Errors in kcal/mol	-24.9	92.3	-6.6	12.4	13.7	2.2	0.1
			Errors in eV	-1.08	4.00	-0.29	0.54	0.59	0.10	0.00
$[Fe(NH_3)_6]^{2+}$	SA-CAS (10,12)	-15.2 [27]	Qu	-1608.597545	-1611.014589	-1611.168797	-1611.168982	-1611.163936	-1611.079206	-1611.085713
			Si	-1608.526007	-1611.135388	-1611.135388	-1611.167507	-1611.165425	-1611.054782	-1611.060012
			Δ_{HS-LS}	-44.90	75.80	-21.00	-0.90	0.90	-15.30	-16.10
			Errors in kcal/mol	-29.7	91	-5.8	14.3	16.1	-0.1	-0.9
			Errors in eV	-1.29	3.95	-0.25	0.62	0.70	0.00	-0.04

S3.4 Optimized Geometries of Iron Complexes

The geometries were taken from Refs. 26 and 28 for the first and the latter two complexes, respectively.

```
Fe(H2O)6^3+
19
Fe   -0.0000000    0.0000000    0.0000000
O     0.0000000    2.0622910    0.0000000
H     0.7919274    2.6471973    0.0000000
H    -0.7919274    2.6471973    0.0000000
O    -0.0000000    0.0000000    2.0622910
H    -0.0000000    0.7919274    2.6471973
H     0.0000000   -0.7919274    2.6471973
O     2.0622910   -0.0000000   -0.0000000
H     2.6471973   -0.0000000    0.7919274
H     2.6471973   -0.0000000   -0.7919274
O    -0.0000000   -2.0622910    0.0000000
H    -0.7919274   -2.6471973   -0.0000000
H     0.7919274   -2.6471973   -0.0000000
O     0.0000000    0.0000000   -2.0622910
H     0.0000000   -0.7919274   -2.6471973
H    -0.0000000    0.7919274   -2.6471973
O    -2.0622910    0.0000000    0.0000000
H    -2.6471973    0.0000000   -0.7919274
H    -2.6471973    0.0000000    0.7919274

Fe(H2O)6^2+
19
Fe   -0.6425718    0.5180392    0.1151526
O     0.1117469    2.1502736   -0.7800331
H     0.7076678    2.7159064   -0.2534686
O    -1.3119384   -0.3025444   -1.5915397
H    -1.9364837    0.1309314   -2.2009076
O     0.0272092    1.3394057    1.8212268
H     0.6525877    0.9068687    2.4304031
O    -1.3964805   -1.1144481    1.0102392
H    -1.9933139   -1.6794048    0.4839749
O    -2.2868202    1.6334083    0.4100537
H    -2.3537702    2.4729107   -0.0830181
O     1.0019437   -0.5971234   -0.1795017
H     1.0680777   -1.4375884    0.3120255
```

```
H    -3.1892210    1.2728025    0.4803642
H    -0.6652199   -0.7835201   -2.1417947
H     1.9045465   -0.2367829   -0.2484504
H     0.4377665    2.1711360   -1.6979435
H    -0.6187560    1.8212395    2.3715987
H    -1.7212601   -1.1359500    1.9285688
```

```
Fe(NH3)6^2+
25
Fe    0.0022693    0.0670392    0.0014801
N     1.7755370    1.1147879    0.0803086
H     1.7233835    2.0198240    0.5640720
H     2.1850067    1.3459344   -0.8329130
H     2.5304149    0.6176911    0.5689385
N     1.0746198   -1.6911361   -0.0836030
H     1.4479323   -2.0197941    0.8154703
H     1.9040384   -1.6473861   -0.6883496
H     0.5498721   -2.4987070   -0.4413837
N    -1.7531423   -1.0113539   -0.0475647
H    -1.8441805   -1.6926662    0.7160240
H    -1.8918420   -1.5751527   -0.8958167
H    -2.6141005   -0.4543296    0.0198086
N    -1.0557297    1.8343475    0.0520904
H    -1.3716833    2.1289166    0.9841643
H    -1.9140027    1.8335855   -0.5123220
H    -0.5368937    2.6495412   -0.2974625
N    -0.0359014   -0.0229493    2.0592111
H    -0.9343371    0.2511934    2.4762670
H     0.6491146    0.5787579    2.5323108
H     0.1383011   -0.9532368    2.4608000
N     0.0096815    0.1786598   -2.0564130
H     0.2630278    1.1003367   -2.4334049
H    -0.8984025   -0.0168361   -2.4959887
H     0.6533168   -0.4672674   -2.5296238
```

References

(1) Stoneburner, S. J.; Truhlar, D. G.; Gagliardi, L. MC-PDFT can calculate singlet–triplet splittings of organic diradicals. *J. Chem. Phys.* **2018**, *148*, 064108.

(2) Sun, Q.; Berkelbach, T. C.; Blunt, N. S.; Booth, G. H.; Guo, S.; Li, Z.; Liu, J.;

McClain, J. D.; Sayfutyarova, E. R.; Sharma, S. et al. PySCF: the Python-based simulations of chemistry framework. *Wiley Interdiscip. Rev. Comput. Mol. Sci.* **2018**, *8*, e1340.

(3) Sun, Q.; Zhang, X.; Banerjee, S.; Bao, P.; Barbry, M.; Blunt, N. S.; Bogdanov, N. A.; Booth, G. H.; Chen, J.; Cui, Z.-H. et al. Recent developments in the PySCF program package. *J. Chem. Phys.* **2020**, *153*, 024109.

(4) Sun, Q.; Zhang, X.; Banerjee, S.; Bao, P.; Barbry, M.; Blunt, N. S.; Bogdanov, N. A.; Booth, G. H.; Chen, J.; Cui, Z.-H. et al. Recent developments in the PySCF program package. *J. Chem. Phys.* **2020**, *153*.

(5) Papajak, E.; Leverentz, H. R.; Zheng, J.; Truhlar, D. G. Efficient Diffuse Basis Sets: cc-pVxZ+ and maug-cc-pVxZ. *J. Chem. Theory Comput.* **2009**, *5*, 1197–1202.

(6) Angeli, C.; Cimiraglia, R.; Malrieu, J.-P. n-electron valence state perturbation theory: A spinless formulation and an efficient implementation of the strongly contracted and of the partially contracted variants. *J. Chem. Phys.* **2002**, *117*, 9138.

(7) Pernal, K.; Hapka, M.; Przybytek, M.; Modrzejewski, M.; Sokół, A. GammCor code. `https://github.com/pernalk/GAMMCOR`, Online; accessed May 20, 2026.

(8) Stoneburner, S. J.; Shen, J.; Ajala, A. O.; Piecuch, P.; Truhlar, D. G.; Gagliardi, L. Systematic design of active spaces for multi-reference calculations of singlet–triplet gaps of organic diradicals, with benchmarks against doubly electron-attached coupled-cluster data. *J. Chem. Phys.* **2017**, *147*, 164120.

(9) Drwal, D.; Beran, P.; Hapka, M.; Modrzejewski, M.; Sokół, A.; Veis, L.; Pernal, K. Efficient adiabatic connection approach for strongly correlated systems: Application to singlet–triplet gaps of biradicals. *J. Phys. Chem. Lett.* **2022**, *13*, 4570–4578.

(10) Saito, T.; Nishihara, S.; Yamanaka, S.; Kitagawa, Y.; Kawakami, T.; Yamada, S.; Isobe, H.; Okumura, M.; Yamaguchi, K. Symmetry and broken symmetry in molecular orbital description of unstable molecules IV: comparison between single- and multi-reference computational results for antiaromtic molecules. *Theor. Chem. Acc.* **2011**, *130*, 749–763.

(11) Schreiber, M.; Silva-Junior, M. R.; Sauer, S. P. A.; Thiel, W. Benchmarks for electronically excited states: CASPT2, CC2, CCSD, and CC3. *J. Chem. Phys.* **2008**, *128*, 134110.

(12) Weigend, F.; Ahlrichs, R. Balanced basis sets of split valence, triple zeta valence and quadruple zeta valence quality for H to Rn: Design and assessment of accuracy. *Phys. Chem. Chem. Phys.* **2005**, *7*, 3297–3305.

(13) Schapiro, I.; Sivalingam, K.; Neese, F. Assessment of n-Electron Valence State Perturbation Theory for Vertical Excitation Energies. *J. Chem. Theor. Comp.* **2013**, *9*, 3567–3580.

(14) Kánnár, D.; Szalay, P. G. Benchmarking coupled cluster methods on valence singlet excited states. *Journal of Chemical Theory and Computation* **2014**, *10*, 3757–3765.

(15) Aidas, K.; Angeli, C.; Bak, K. L.; Bakken, V.; Bast, R.; Boman, L.; Christiansen, O.; Cimiraglia, R.; Coriani, S.; Dahle, P. et al. The Dalton quantum chemistry program system. *WIREs Comput. Mol. Sci.* **2014**, *4*, 269–284.

(16) Goll, E.; Werner, H.-J.; Stoll, H. A short-range gradient-corrected density functional in long-range coupled-cluster calculations for rare gas dimers. *Phys. Chem. Chem. Phys.* **2005**, *7*, 3917–3923.

(17) Goll, E.; Werner, H.-J.; Stoll, H.; Leininger, T.; Gori-Giorgi, P.; Savin, A. A short-range gradient-corrected spin density functional in combination with long-range coupled-cluster methods: Application to alkali-metal rare-gas dimers. *Chem. Phys.* **2006**, *329*, 276–282.

(18) Paziani, S.; Moroni, S.; Gori-Giorgi, P.; Bachelet, G. B. Local-spin-density functional for multideterminant density functional theory. *Phys. Rev. B* **2006**, *73*, 155111.

(19) Schapiro, I.; Sivalingam, K.; Neese, F. Assessment of n-electron valence state perturbation theory for vertical excitation energies. *J. Chem. Theory Comput.* **2013**, *9*, 3567–3580.

(20) Werner, H.-J.; Knowles, P. J.; Knizia, G.; Manby, F. R.; Schütz, M. Molpro: a general-purpose quantum chemistry program package. *Wiley Interdiscip. Rev.: Comput. Mol. Sci.* **2012**, *2*, 242–253.

(21) Balabanov, N. B.; Peterson, K. A. Basis set limit electronic excitation energies, ionization potentials, and electron affinities for the 3d transition metal atoms: Coupled cluster and multireference methods. *J. Chem. Phys.* **2006**, *125*.

(22) De Jong, W. A.; Harrison, R. J.; Dixon, D. A. Parallel Douglas–Kroll energy and gradients in NWChem: Estimating scalar relativistic effects using Douglas–Kroll contracted basis sets. *J. Chem. Phys.* **2001**, *114*, 48–53.

(23) Reiher, M.; Wolf, A. Exact decoupling of the Dirac Hamiltonian. I. General theory. *J. Chem. Phys.* **2004**, *121*, 2037–2047.

(24) Knizia, G. Intrinsic Atomic Orbitals: An Unbiased Bridge between Quantum Theory and Chemical Concepts. *J. Chem. Theory Comput.* **2013**, *9*, 4834–4843.

(25) Knizia, G. Intrinsic Bond Orbitals: An Unbiased Representation of Chemical Bonding. *J. Chem. Theory Comput.* **2013**, *9*, 4834–4843.

(26) Radoń, M. Benchmarking quantum chemistry methods for spin-state energetics of iron complexes against quantitative experimental data. *Phys. Chem. Chem. Phys.* **2019**, *21*, 4854–4870.

(27) Domingo, A.; Àngels Carvajal, M.; De Graaf, C. Spin crossover in Fe (II) complexes: An ab initio study of ligand σ-donation. *Int. J. Quant. Chem.* **2010**, *110*, 331–337.

(28) Floser, B. M.; Guo, Y.; Riplinger, C.; Tuczek, F.; Neese, F. Detailed pair natural orbital-based coupled cluster studies of spin crossover energetics. *J. Chem. Theory Comput.* **2020**, *16*, 2224–2235.
